# Cardiovascular Imaging for Systemic Sclerosis Monitoring and Management

**DOI:** 10.3389/fcvm.2022.846213

**Published:** 2022-03-31

**Authors:** Peter Glynn, Sarah Hale, Tasmeen Hussain, Benjamin H. Freed

**Affiliations:** ^1^Division of Cardiology, Department of Medicine, Northwestern University Feinberg School of Medicine, Chicago, IL, United States; ^2^Division of Hospital Medicine, Department of Medicine, Northwestern University Feinberg School of Medicine, Chicago, IL, United States

**Keywords:** cardiovascular imaging, systemic sclerosis (scleroderma), echocardiography, cardiovascular magnetic resonanace, scleroderma heart disease

## Abstract

Systemic sclerosis (SSc) is a complex connective tissue disease with multiple clinical and subclinical cardiac manifestations. SSc can affect most structural components of the heart, including the pericardium, myocardium, valves, and conduction system through a damaging cycle of inflammation, ischemia, and fibrosis. While cardiac involvement is the second leading SSc-related cause of death, it is frequently clinically silent in early disease and often missed with routine screening. To facilitate identification of cardiac disease in this susceptible population, we present here a review of cardiac imaging modalities and potential uses in the SSc patient population. We describe well-characterized techniques including electrocardiography and 2D echocardiography with Doppler, but also discuss more advanced imaging approaches, such as speckle-tracking echocardiography, cardiovascular magnetic resonance imaging (CMR), and stress imaging, among others. We also suggest an algorithm for the appropriate application of these modalities in the workup and management of patients with SSc. Finally, we discuss future opportunities for cardiac imaging in SSc research to achieve early detection and to optimize treatment.

## Introduction

**FIGURE 1:**
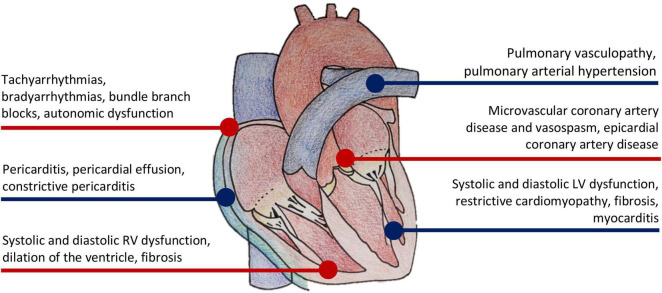
Manifestations of cardiovascular disease in systemic sclerosis.

Cardiac complications from systemic sclerosis (SSc) are numerous and often underdiagnosed. These complications are either a direct result of SSc on the myocardium, or indirect effects of other organ involvement [pulmonary arterial hypertension (PAH), interstitial lung disease (ILD), or renal crisis, etc.) (1). Direct myocardial manifestations of SSc include microvascular coronary artery disease, cardiac fibrosis, myocarditis, left and right ventricular systolic and diastolic dysfunction, pericardial disease, and conduction abnormalities (Figure 1) (2). When present, cardiac involvement predicts worse survival (3). In two meta-analyses of observational studies, cardiac deaths represented the most frequent cause of death in SSc, accounting for 20–29% of all deaths (4, 5). Early screening and detection of cardiac abnormalities is thus vitally important in SSc. Cardiac imaging is utilized extensively for that purpose.

As cardiac imaging techniques have progressed, they have allowed for new insights into the pathophysiology, diagnosis, and treatment of SSc. While two-dimensional echocardiography (2DE) remains the most utilized modality, tissue doppler imaging (TDI), speckle-tracking imaging (STE), and cardiac magnetic resonance imaging (CMR) all have growing importance. In the present review, we will discuss the most recent data regarding the epidemiology, diagnosis, and management of cardiac complications of SSC, highlighting the evolving role that cardiac imaging plays in diagnosis and management.

## Epidemiology

The prevalence of cardiac involvement in SSc is difficult to define for a number of reasons – rates vary depending on the methods used, signs and symptoms of cardiac disease can be subtle leading to underestimation, and symptoms of cardiac involvement are often attributed to pulmonary, musculoskeletal, or esophageal involvement. Rates of cardiac involvement vary depending on which manifestation is being considered. Broadly speaking, rates range from as low as 7% to as high as 44% depending on the study (6, 7). Major risk factors for cardiac involvement include male sex, African-American ethnicity, diffuse cutaneous SSc, older age at disease onset, tendon friction rubs, abnormal nail-fold capillaroscopy, and worse quality-of-life scores (6).

## Right Ventricular Dysfunction and Pulmonary Hypertension

In patients with SSc, right ventricular (RV) dysfunction is more common than left ventricular (LV) dysfunction and may exist due to primary myocardial involvement, increased pulmonary vascular resistance (PVR), or a mixture of both (2, 8, 9). RV function may occur early in the disease course (2, 9, 10) and myocardial fibrosis may be responsible for both diastolic and systolic dysfunction (2, 8, 11). While RV dysfunction is often associated with abnormal pulmonary artery (PA) pressures and PAH, myocardial fibrosis may directly lead to RV dysfunction and elevated right atrial pressure (RAP) even in the absence of elevated PA pressures (11).

In patients with SSc, pulmonary hypertension (PH) may manifest through various pathologic mechanisms, and can be categorized into one or more of the five WHO Groups of PH. SSc associated-PAH (SSc-PAH) is considered WHO Group 1 PH and may be the result of direct remodeling of the pre-capillary pulmonary arterioles, or rarely be attributable to pulmonary veno-occlusive disease/pulmonary capillary hemangiomatosis (PVOD/PCH). It is a leading cause of mortality in SSc patients, with an incidence of 8–13% (12, 13) and is more commonly associated with limited cutaneous systemic sclerosis (lcSSc) (14), Patients with SSc-PAH have a poor prognosis in comparison to SSc patients without PAH, with 3 year survival of only 52% (14–16). Several studies have demonstrated that the intrinsic RV dysfunction in SSc patients leads to worse outcomes in SSc-PAH in comparison to idiopathic PAH (2, 13, 17). One year mortality for SSc-PAH has been found to be 30%, while one year mortality for those with idiopathic PAH is 15% (17). Mortality rates remain high despite therapy (14).

Systemic sclerosis associated PH can also occur in the setting of capillary loss and hypoxemic respiratory failure as a result of ILD, categorized as WHO Group 3 PH. PH due to ILD is more common in patients with diffuse cutaneous systemic sclerosis (dcSSc) (18). In addition, patients with SSc may have evidence of left ventricular systolic and diastolic dysfunction, leading to WHO Group 2 PH. Rarely, patients with SSc may develop chronic thromboembolic pulmonary hypertension (CTEPH) and be classified as WHO Group 4 PH. Distinguishing among the different mechanisms of SSc associated PH is important as treatment and prognosis are impacted by a timely and accurate diagnosis (14, 19, 20).

In recent years, there has been a greater focus on understanding the pathophysiologic mechanisms driving RV dysfunction and PH associated with SSc patients, as well as the poor outcomes linked to the presence of PH in this patient population. Essential to this focus is the thorough investigation of the etiology of PH in patients with SSc, which often includes diagnostic tests such as full pulmonary function testing, including diffusion capacity for carbon monoxide (DLCO) for PH-ILD, ventilation perfusion scanning (V/Q scan) for CTEPH, and computed tomography scanning for radiographic correlates of PVOD/PCH (19). Cardiac imaging is frequently used for this purpose as well as diagnosing and monitoring disease status. 2DE and cardiovascular magnetic resonance (CMR) are the most common imaging modalities used. Notably, STE is a novel way to detect RV dysfunction earlier and its role in SSc associated RV dysfunction and PH is currently being studied. It is important to screen patients for PH and RV dysfunction early, as studies have demonstrated improved survival and improved treatment response in SSc-PAH patients with mild hemodynamics and symptoms (14, 21).

### Evaluation

Given the high prevalence of RV dysfunction and PH in patients with SSc, current guidelines recommend echocardiographic screening of all SSc patients in order to detect and implement PH therapy as early as possible (22). In select patients, further imaging with CT or CMR may be helpful.

### Echocardiography

Transthoracic echocardiography (TTE) is widely available and accessible to patients, making it an optimal screening tool for RV dysfunction and PH. Evidence of RV dysfunction on 2DE include RV dilation, flattening of interventricular septum (D-Sign) suggestive of RV pressure or volume overload, and reduced semi quantitative measures of RV function [i.e., tricuspid annular systolic planar excursion (TAPSE) and fractional area of change (FAC)]. Significant RV dysfunction has been defined as TAPSE less than 1.7 cm and FAC less than 35% (10).

Transthoracic echocardiography can also be used to distinguish WHO Group 1 PH from WHO Group 2 PH in patients with SSc. For example, echocardiographic findings suggestive of left heart disease, such as left atrial dilatation, abnormal markers of diastolic dysfunction (i.e., elevated E/e’ ratio, reduced mitral annular tissue doppler velocities), or evidence of LV dysfunction, may be indicative of post-capillary PH (WHO Group 2 PH). On the other hand, echocardiographic markers demonstrating elevated PA pressures, RV dilation, and/or RV dysfunction, in the absence of left heart disease, is more suggestive of pre-capillary PH (WHO Group 1 PH). Additional measures such as RV outflow tract (RVOT) systolic doppler flow velocity notching and RVOT acceleration time may also be useful in differentiating pre- versus post-capillary PH (23).

Right atrial and RV chamber enlargement in SSc are directly related to onset of heart failure symptoms as well as mortality (10). In addition, measurements of RV systolic function such as TAPSE, tissue Doppler of the tricuspid annulus S’, RV FAC, and Tei index all correlate with decreased survival (10). Argula et al. demonstrated an improvement in TAPSE after medical therapy in patients with IPAH, while patients with SSc-PAH had worsening tricuspid regurgitant jet velocity and larger right heart chamber size after therapy (24). The attenuated response to PH therapy in the SSc population, demonstrated through echocardiographic markers, may signal the presence of intrinsic RV dysfunction in SSc-PAH patients in comparison to IPAH patients (13, 25).

Both RV systolic and diastolic dysfunction are prevalent in SSc patients (8). When compared to controls, Muene et al. showed that SSc patients had significantly reduced RV contractility (*p* < 0.001) and larger RA area. In addition, 25% of SSc patients had abnormal RV diastolic dysfunction compared to 0% in controls, as measured by trans-tricuspid E/A ratio (8). Although patients with SSc-PAH demonstrate greater prevalence of reduced RV contractility and diastolic dysfunction, patients with SSc who did not meet diagnostic criteria for PAH still had abnormalities in both RV systolic and diastolic dysfunction (8).

Abnormal echocardiographic findings can also be demonstrated early in the disease course, even before signs or symptoms manifest themselves (26). In a study by Pigatto et al., 45 SSc patients without any signs or symptoms of heart disease or PH were compared to 43 healthy controls, using both 2DE and three-dimensional echocardiography (3DE). The SSc patients were found to have a significant increase in RV volume and reduced RV ejection fraction as determined by 3DE. Doppler measurements demonstrated an increased systolic pulmonary artery pressure (sPAP) in SSc patients in comparison to the control group. In addition, the changes were more pronounced in patients with lcSSc than dcSSc (26). This study underscores the need for screening for RV dysfunction and PH in this patient population even in the absence of symptoms.

### Speckle Tracking Echocardiography

Although RV dysfunction and PH in SSc is common and is a large determinant of long term prognosis, it often remains undetectable despite the use of 2D echocardiography. Most 2DE measures of RV function are limited by the complex shape of the RV, load dependency, and suboptimal reproducibility. STE avoids some of these pitfalls and can be used to detect subtle changes in global and regional RV systolic function that may be undetectable by conventional echocardiographic measures (10).

**FIGURE 2:**
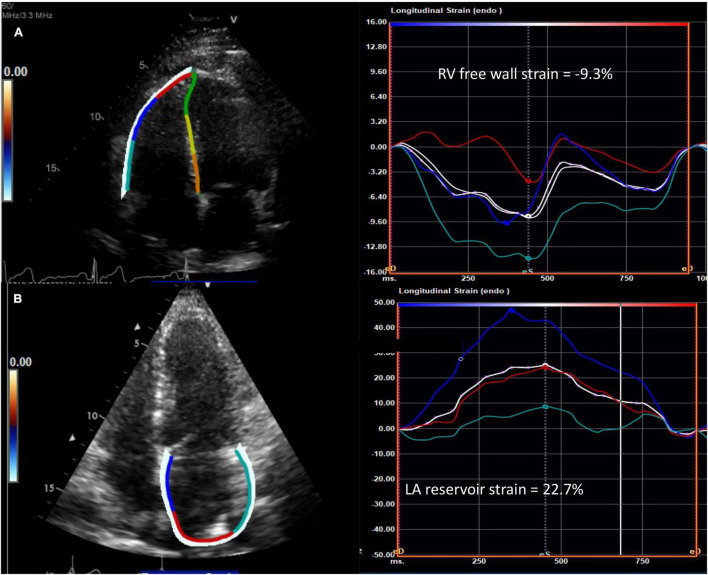
Abnormal speckle tracking strain in diffuse cutaneous systemic sclerosis. **(A)** Example of reduced right ventricular free wall strain (normal <-20%) and **(B)** reduced left atrial reservoir strain (normal >39%) from the apical four chamber view in patients with systemic sclerosis. The white curve represents the average of the peak systolic strain curves. RV, right ventricle; LA, left atrium.

RV longitudinal systolic strain (RVLSS) has been shown to be more abnormal in SSc when compared to controls, despite comparable traditional echocardiographic parameters of RV function between groups (Figure 2) (27). For example, a study by Zairi et al. revealed a 3.3 fold increased risk of subclinical RV systolic impairment in SSc patients, manifested by more abnormal RVLSS, in comparison to controls (-18.2 vs. 22.3%; *p* = 0.012) (27). Meanwhile, multiple authors have illustrated a regional pattern of abnormal RVLSS seen in SSc patients (10, 28, 29). In a study by Mukherjee et al., 138 SSc patients were compared to 40 healthy non-SSc controls. While both TAPSE and RV FAC were normal in both groups, RVLSS was found to be abnormal in SSc patients, independent of PH and SSc phenotype. Specifically, a regional pattern of abnormal strain was seen in SSc patients, with increased strain in the basal segment and decreased strain in the apical and mid segments. Some hypothesize that the basal segment is hyperkinetic early in the disease course, and as PH develops, the ability of the basal segment to compensate decreases and RV failure ensues, suggesting a “two hit” hypothesis in which pre-existing RV contractile dysfunction may predispose to further dysfunction after PH occurs (28). While STE is a promising technique for detecting unique patterns of early, subclinical RV dysfunction in SSc patients, more evidence is needed to discern its role in this population.

### Exercise Echocardiography

Recent studies have investigated whether exercise echocardiography can unmask PH in SSc patients potentially leading to earlier diagnosis and therapy for patients with SSc-associated PH. A study by Rallidis et al. investigated 49 patients with SSc undergoing exercise stress echocardiography, and found that post-exercise TR velocity > 3.4 m/s had a sensitivity of 90.5% and a specificity of 80% in detecting PH, as confirmed with right heart catheterization (RHC) (30). In addition, a study by Mukherjee et al. used STE to demonstrate that SSc-PAH patients had diminished RV contractile reserve in response to exercise (29). The authors demonstrated that all patients with SSc had regional abnormalities in RVLSS at rest, but patients with elevated RV systolic pressure (defined by RV systolic pressure ≥35 mmHg) lacked an ability to increase both global and regional strain with exercise. This finding suggests a lack of RV contractile reserve in these patients likely due to intrinsic myocardial dysfunction (29).

Although exercise-induced PH has clinical and prognostic significance in patients with cardiopulmonary conditions, there remains a lack of standard definition for exercise-induced PH and a lack of standard measures for how the RV and pulmonary vasculature respond to exercise (14). Further study is needed to determine the utility of exercise echocardiography in unmasking PH, as well as the use of exercise echocardiography in assessing RV and PV contractile reserve.

### Cardiovascular Magnetic Resonance

**FIGURE 3:**
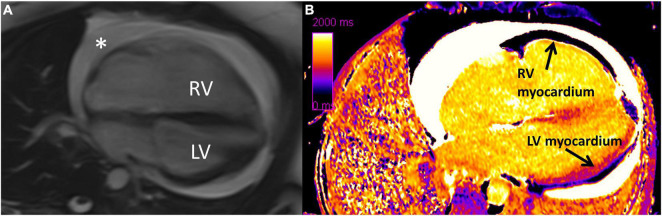
Diffuse interstitial fibrosis of the right ventricle using cardiovascular magenetic resonance imaging. **(A)** Four chamber cine showing enlarged right ventricle and circumferential pericardial effusion (*). **(B)** Native T1 map showing increased T1 time (diffuse fibrosis) in right ventricular myocardium compared to left ventricular myocardium. RV, right ventricle; LV, left ventricle. Note: this image is reproduced with permission from Belin et al. (11).

Cardiovascular magnetic resonance is the gold standard for measuring RV size and function and is superior in terms of reproducibility to 2DE in that it does not require a suitable acoustic window for measuring RV size, geometry, and function. CMR cine imaging provides excellent spatial resolution and free breathing sequences provides real-time physiologic assessment of the interventricular septal dynamics. While some interventricular septal flattening is expected during inspiration to accommodate the increased volume of blood, significant interventricular septal flattening is indicative of a dysfunctional RV. Although evaluation of myocardial fibrosis with late gadolinium enhancement (LGE) is useful for the LV, it is challenging with the RV due to the RV’s thin myocardium. Therefore, high resolution T1 mapping and myocardial extracellular volume (ECV) have emerged as useful techniques in detecting diffuse RV fibrosis, even in the absence of PH. CMR measures of myocardial fibrosis, such as T1 mapping and ECV, are valuable to detect fibrosis early in the disease course, which can potentially alter therapy and prevent worsening RV dysfunction (Figure 3) (2, 11, 31–36). A study by Chaosuwannakit et al. found that 21% of patients with SSc who underwent CMR had RV dilation despite not meeting criteria for PH suggesting the presence of intrinsic RV dysfunction – perhaps due to fibrosis – in these patients (37). It is hypothesized that myocardial fibrosis reflects a cellular response to increased RV afterload, but more investigation is needed to understand if myocardial fibrosis on CMR signals an adaptive or maladaptive response (35).

While STE is shown to be beneficial in assessing subclinical RV dysfunction in SSc-associated PH, CMR-derived strain has also exhibited utility in assessing patients’ response to PH therapy (38). Measurements of strain and strain rate can be obtained using tissue-tracking CMR. In the ATPAHSS-O trial, 21 treatment naïve patients with SSc were analyzed using pre and post-treatment CMR examinations. The study found a significant improvement in measures of RVLSS, RV peak systolic longitudinal strain rate, RV peak longitudinal atrial-diastolic strain rate, and RV peak circumferential early diastolic strain rate after 36 weeks of treatment. Notably, improvements in RV and LV strain also correlated with improvements in clinical outcomes (38).

### Computed Tomography

When evaluating a SSc patient using CT, increased PA diameter (>29 mm), septal flattening, and increased RV-LV ratio suggest elevated pulmonary artery pressures (2, 22). High resolution CT (HRCT) is used to assess for interstitial lung disease and for signs of PVOD that can complicate SSc-PH diagnosis and therapy (2, 39). Interstitial changes are visible on HRCT in up to 80% of SSc patients, while ILD is clinically apparent in up to 40% of patients (14). While ILD is common in SSc, there is no validated definition of the optimal threshold of lung involvement to differentiate SSc-PH associated with ILD from SSc-PAH (14). It has been demonstrated that combined fibrosis and emphysema is associated with an increased risk of PH (14, 39–41), and several studies have demonstrated poorer survival with SSc-PH associated with ILD compared to SSc-PAH, making an accurate diagnosis important (42, 43).

### Right Heart Catheterization

Right heart catheterization remains the gold standard for hemodynamic assessment of PH (2). Per the 2015 ESC/ERS Guidelines for the diagnosis and treatment of pulmonary hypertension (22), the decision to pursue a RHC depends on three scores which include (1) peak tricuspid regurgitation velocity; (2) the presence of at least 2 PH signs on TTE [from two different categories among (a) the ventricles, (b) the pulmonary artery, and (c) the inferior vena cava and right atrium]; and (3) the presence of CTEPH or PAH risk factors or associated conditions. For patients with high echocardiographic probability of PH based on echo criteria (1 and 2 above), RHC is recommended. When the probability of PH is intermediate by echo but CTEPH or PAH risk factors are present, RHC should be considered. In patients with symptoms such as exertional dyspnea, angina, syncope, and exercise intolerance that remain unexplained after initial cardiopulmonary evaluation, RHC should be considered, especially when SSc is suspected or confirmed.

Patients are often first screened with echocardiography, and recommendations suggest referral for RHC if the tricuspid regurgitant velocity is more than 2.8 m/s, or more than 2.5 m/s if signs or symptoms of PH are present (2, 44). Because patients with SSc can develop PH through a variety of pathophysiologic mechanisms, RHC is recommended for confirmation of elevated PA pressures on 2DE and to differentiate between different mechanisms of PH (2, 22).

## Left Ventricular Dysfunction

Left ventricular dysfunction in systemic sclerosis is most likely a result of microvascular disease and inflammation. Most patients do not have appreciable CAD (45). Autopsies of myocardial tissue from SSc patients show focal areas of pathology, ranging from contraction band necrosis to replacement fibrosis in the tissue (45). Early detection of fibrosis may assist clinicians in identifying those SSc patients at risk of arrythmia, rehospitalization, and cardiovascular mortality (46).

Left ventricular involvement in SSc includes primarily diastolic dysfunction, and, in some cases, restrictive cardiomyopathy. Several studies show that overtly decreased left ventricular ejection fraction (LVEF) is a relatively uncommon feature of SSc (7, 47–50). Non-invasive identification of LV abnormalities through imaging may have substantial diagnostic as well as prognostic benefits to clinicians and patients, as further elucidated below.

### Evaluation

Echocardiography in SSc remains the best initial tool for the evaluation of LV disease, due to its low cost, lack of radiation, and extensive evidence base, as noted below. Tissue Doppler and STE, specifically, are sensitive tests for uncovering subclinical LV dysfunction, which may be suggestive of microvascular disease. Guidelines recommend asymptomatic patients with SSc receive yearly echocardiography by trained sonographers following a standardized Scleroderma Doppler Echocardiography Protocol, which includes multiple standardized 2D echo and Doppler images (51). In select patients, further imaging with CT or cardiac MRI may be helpful, though the literature is less clear on specific recommendations regarding these modalities.

### Echocardiography

Compared to healthy patients, patients with SSc undergoing echocardiography show impaired LV diastolic function noted by multiple measures. Structurally, LV hypertrophy appears more common in SSc than controls (52). Patients with SSc also have higher E/e’ ratios (53) (representing higher LV filling pressures) and increased isovolumetric relaxation times compared to controls (48). Interestingly, abnormal E/e’ ratio [defined as transmitral to mitral annular early diastolic velocity ratio (54)] was also associated with duration of recognized SSc disease in months, as well as mean duration of Raynaud’s phenomenon, suggesting temporal relation in SSc-related fibrotic disease of different organs (49). Additionally, certain measurements may also predict mortality in SSc patients. One study utilizing lateral tissue Doppler early mitral annular (e’) velocity found that each standard deviation decrease in e’ (suggesting impaired LV relaxation) was associated with increased risk of death in SSc patients at 1.9 ± 1.3 years (55). Thus, 2DE with tissue Doppler analysis can provide important structural clues that can correlate to disease progression as well as provide important prognostic information.

### Speckle Tracking Echocardiography

Systemic sclerosis patients also show differences in strain imaging via echocardiography as compared to controls. Studies have shown both regional and global LV longitudinal systolic strain (LVLSS) is significantly lower in SSc patients than controls and is most pronounced in the endocardial layer of the LV (56, 57). Furthermore, SSc patients with lower global LVLSS were more likely to have elevated C-reactive protein values suggesting greater inflammation. LVLSS also appears to worsen in SSc patients over time (56, 58).

Lindholm et al. showed that SSc patients with PAH had the lowest LVLSS when compared to both SSc patients without PAH as well as controls, suggesting that PAH may be a co-contributor to apparent LV disease in this population (59). Similarly, SSc patients with hypertension had a higher prevalence of diastolic dysfunction and worse LVLSS when compared to SSc patients without hypertension and controls with and without hypertension (60). Thus, STE can help identify subclinical cardiac disease in SSc, and can also be used to show the incremental effects of other medical conditions on an already vulnerable myocardium.

### Cardiovascular Magnetic Resonance

Similar to RV pathology, CMR provides information on LV dysfunction beyond echocardiography in patients with SSc. In a study evaluating 41 patients with SSc, Tzelepis et al. identified mesocardial LGE in a linear pattern located in the basal and midcavity segments of the LV. Patients with a greater than 15-year duration of Raynaud’s phenomenon had a greater number of enhancing segments than those with a shorter duration of Raynaud’s. Furthermore, those with abnormal Holter monitor results [defined as conduction delay or block, intermittent bundle branch block, ventricular arrythmias (mono- or polymorphic, or >100/day premature ventricular contractions {PVCs}), or ventricular tachycardias] over 24 h also had a greater number of enhancing segments suggesting an association between degree of fibrosis and arrhythmias (61).

As mentioned earlier, ECV can identify diffuse fibrosis even in patients with normal echocardiograms and without any evidence of LGE on CMR. Thuny et al. showed that SSc patients with normal echocardiograms had significantly higher global LV ECV than age matched healthy controls and that global LV ECV significantly correlated with the grade of diastolic dysfunction (62). Another study found that, in addition to higher ECV, SSc patients also had less myocardial blood flow augmentation (measured by CMR perfusion imaging) when experiencing a cold pressor test (hand immersion in cold water) compared to healthy controls. This muted vasodilatory response was favored to represent microvascular dysfunction in this population (63). Higher baseline ECV also correlates to risk of cardiovascular events among SSc patients with normal echocardiograms who are monitored over time. In a study of 50 patients with dcSSc, Markousis-Mavrogenis et al. found that baseline LGE, LV mass, T2 mapping (a measure of edema) and ECV values were all significant predictors of CV complications (arrythmia, heart failure, pulmonary hypertension, and/or sudden cardiac death) over 1.2 years, including when data were controlled for sex, age, and duration of disease (64).

Similar to stress echocardiography, stress CMR may be more sensitive than rest CMR in detecting subclinical disease. Asymptomatic patients with SSc show evidence of significantly decreased myocardial perfusion on adenosine stress CMR when compared to controls, even when there is no difference on rest CMR (65). An additional study found that, of those patients with stress CMR perfusion defects, none had correlating stenotic lesions on coronary CT, suggesting that microvascular disease, rather than epicardial CAD, may be the primary driver of hypoperfusion in SSc (66).

## Pericardial Disease

Pericardial disease is a common feature of scleroderma, with manifestations including pericardial inflammation, effusion, fibrinous pericarditis, pericardial adhesions, and rarely constrictive pericarditis or tamponade (1, 67). The prevalence of clinically apparent pericardial disease is 5–16%, but autopsy studies suggest the incidence of asymptomatic involvement may be higher (1). Contemporary imaging studies suggest the incidence of pericardial effusion is between 15 and 19% (68, 69). Large effusions and those complicated by tamponade are rare but associated with poor outcomes (70). Drainage of these effusions or creation of a pericardial window in the context of associated PAH is associated with significant mortality (70, 71). Constrictive pericarditis represents a challenging diagnosis in this population as clinical symptoms may not be present until right-heart failure develops, and symptoms may overlap with signs of PAH and SSc-associated cardiomyopathy. For this reason, among others, a multimodality imaging approach is important in the assessment of pericardial disease (72).

### Evaluation

The diagnostic approach for patients with pericardial disease and SSc is the same for those without associated SSc. All patients should receive an electrocardiogram and transthoracic echocardiogram. In select patients, further imaging with CT or CMR may be helpful.

### Electrocardiography

In acute pericarditis, the EKG may demonstrate classic findings such as diffuse ST segment elevations and PR segment depressions, but up to 40% present with atypical and non-diagnostic findings (73). For those with small to moderate pericardial effusions, there may be no EKG changes. In large pericardial effusions, low voltage and electrical alternans may be present (74).

### Echocardiography

2DE is considered the first line imaging modality in almost all types of pericardial disease because it is safe, readily available, and quick to perform (74). 2DE allows for detection of pericardial effusion and assessment of effusion size. It also can assess for hemodynamic features of tamponade, including diastolic collapse of right-sided chambers, significant respirophasic changes across the mitral and tricuspid valves, and ventricular interdependence with abnormal septal bounce during inspiration (75). Tissue Doppler imaging is especially helpful in the diagnosis of constrictive pericarditis. A high early (E) velocity, shortened deceleration time, and reduced atrial (A) wave are characteristic. With inspiration, mitral inflow velocity typically falls by 25–40%, while tricuspid velocity increases by 40–60% (75). STE may also be useful in this assessment, with reduced circumferential strain and preserved global longitudinal strain consistent with constriction (75). In addition, 3DE is useful in better delineating the extent of pericardial thickening and the exact size, location, and extent of stranding within the pericardial effusion (76). Because many patients with acute pericarditis may have a normal 2DE (75), additional imaging modalities such as CMR or CT may be helpful if clinical suspicion is high.

### Computed Tomography

Computed tomography attenuation of the pericardium is similar to the myocardium so visualization of the pericardium can be challenging on CT. However, pericardial calcifications are well visualized on CT. CT density measurements facilitate the characterization of pericardial fluid; low density fluid [0–20 Hounsfield Units (HU)] is typical, while hemorrhagic effusions or those associated with bacterial infections may have densities of 50 HU or more (74). Because of the similar attenuation of myocardium and pericardium on CT, echocardiography and CMR are typically preferred.

### Cardiovascular Magnetic Resonance

Due to its high inherent tissue contrast, excellent spatial and temporal resolution, and ability to reconstruct in multiple planes, CMR is well-suited for the evaluation of pericardial disease (77). While not necessary for the diagnosis of acute pericarditis, CMR can be helpful for those with incessant (ongoing symptoms >4–6 weeks, but <3 months), recurrent, or chronic (>3 months) of symptoms, or those in which clinical suspicion is high but initial evaluation (EKG, echo) has been negative. Pericardial thickening is readily viewed, and pericardial edema and inflammation can be evaluated by both T2-weighted LGE imaging (77). Fat suppression can increase the specificity of these findings. CMR has been used to guide steroid therapy in recurrent pericarditis, leading to lower overall steroid doses without an increased risk of constrictive pericarditis or need for pericardial window, and with lower rates of recurrence (78).

Constrictive pericarditis typically presents with thickening of the pericardium, which is best appreciated on T1-weighted imaging. However up to 18% of patients may have normal pericardial thickness (79). Pericardial fusion in the absence of active inflammation and parietal-visceral adherence are indicative of constrictive pericarditis (80, 81). Hemodynamic indicators of constriction are also well visualized on CMR; respiratory flow variation across the mitral valve greater than 25% is sensitive and specific for constriction, as is increased relative septal excursion (82, 83). While echocardiography remains the standard, consider CMR imaging when concern for pericarditis is high but echocardiography is unrevealing, or when symptoms of pericarditis persist or recur over months.

### Catheterization

Invasive hemodynamic measurement of right and left ventricular pressure measurement is also important in the assessment of constriction. The “dip and plateau” sign (end-diastolic equalization of pressures), while not specific to constriction, can help confirm the diagnosis in the right clinical context (74).

## Arrhythmia

Electrophysiologic studies of patients with SSc report conduction defects, arrhythmia, and autonomic dysfunction in up to 51% of patients (6). Atrial and ventricular ectopy, atrial fibrillation and flutter, supraventricular tachycardia, ventricular tachycardia, and atrioventricular block have all been associated (6). In a recent study from a Danish cohort, there was a nearly two-fold relative risk increase for incident atrial fibrillation and flutter as well as pacemaker or ICD placement in patients with SSc compared to age-matched controls (84). In the EUSTAR database, arrhythmias caused 6% of SSc-related deaths, behind only pulmonary fibrosis and PAH (85). The mechanism of this is thought to be multifold- the direct consequences of microvascular injury, the effects of fibrosis in the conduction system and myocardium, and autonomic dysfunction (51, 86). Importantly, autonomic dysfunction occurs early in the disease course, even before the development of fibrosis or other visceral manifestations. Low heart rate variability is a marker of autonomic dysfunction and is correlated with pre-clinical cardiac involvement (86).

### Evaluation

Arrhythmia is assessed by EKG and cardiac monitoring as indicated. 2DE and CMR can be useful adjuncts to detect structural or functional disease that may contribute to the arrhythmia.

### Electrocardiography

Screening EKG should be performed in all patients with SSc (51). While not a particularly sensitive test, this can help identify both conduction abnormalities and signs of hypertrophy as well as chamber enlargement. Right bundle branch block has been associated with a greater than five-fold increase in mortality risk, thought to be reflective of either underlying lung pathology or cardiac involvement (87). In a study of 100 SSc patients investigated with new onset heart failure, 56% had EKG abnormalities and 24% had PVCs. The PVC burden corresponded positively with high-sensitivity troponin and negatively with LV ejection fraction. Seven patients in this study either suffered sudden cardiac death or required ICD placement; the presence of more than 1190 PVCs/day identified these patients with a sensitivity of 100% and specificity of 83% (88). Prolonged QTc interval and QT dispersion may also predict ventricular arrhythmias, as might an abnormal signal-averaged EKG (SAE) (89, 90). Holter monitoring should be utilized if there is any clinical concern for conduction abnormality, and there should be a low threshold for implantable loop recorder placement in the appropriate clinical context (51).

### Echocardiography and Cardiovascular Magnetic Resonance

All patients with scleroderma should receive a routine screening echo, regardless of the presence or absence of arrhythmia. The recently published Scleroderma Arrhythmia Clinical Utility Study (SAnCtUS) evaluated 150 consecutive SSc patients assessed with 24-h Holter and CMR, looking at markers of LV function, edema (T2 mapping) and fibrosis (LGE). T2 mapping and LGE were significant predictors for ventricular arrhythmias, but not supraventricular arrhythmias. Using these CMR variables, the study developed the SAnCtUS score to predict risk of ventricular arrhythmia. The authors found that those with the highest score, independent of ejection fraction or the presence of ventricular tachycardia on baseline Holter, were 3.86 times more likely to have sustained ventricular tachycardia or sudden cardiac death at one year compared to those with lower scores (91). These data suggest a possible role for CMR to risk stratify patients for life threatening arrhythmias in the future, but this requires further evaluation and prior to incorporation into routine clinical care.

As evident in the discussion above, cardiac imaging modalities offer different but often complementary data in the evaluation of the SSc patient. Understanding the strengths and weaknesses of each diagnostic test is necessary when deciding how best to manage a particular symptom or abnormal finding. We have summarized the utility of each modality, along with their strengths and weaknesses, for the most common cardiac complications of SSc in Table 1.

**TABLE 1:** Applications, strengths, and weaknesses of common diagnostic modalities in scleroderma heart disease.

	Imaging Modalities			Adjunctive Modalities	
SSc Disease Process	Echo	CT	CMR	EKG	RHC
RV Dysfunction	Initial screening tool. Assesses for: ● chamber enlargement ● diastolic dysfunction (TV E/A) ● systolic function (TAPSE, FAC, RVEF by 3DE) ● RV hemodynamics (RVSP) ● subclinical disease (strain, exercise stress echo)	Often in conjunction with pulmonary imaging. Assesses for: ● Chamber size, hypertrophy ● RVEF ● RV pressure and volume overload (septal flattening, increased RV-LV ratio)	Gold standard for assessing RV given complicated 3D geometry. Assesses for: ● chamber enlargement ● diastolic dysfunction (RV mass, hypertrophy) ● systolic function (RVEF) ● RV hemodynamics (RVSP) ● subclinical disease (strain) ● fibrosis and edema (T1 mapping, T2 mapping, ECV)	Signs of RVH, RV strain	Direct measurement of RV systolic and diastolic pressure, assessment of cardiac output
PAH	Initial screening tool. Provides estimates of PASP based on TV regurgitant jet velocity.	Cannot directly estimate pressure but may demonstrate increased PA diameter and signs of RV strain.	Like echo, provides estimates of PASP using estimates of TV regurgitant jet velocity.	Signs of RVH, RV strain	Gold standard for hemodynamic assessment of PH. Allows for differentiation among WHO groups
LV Dysfunction	Initial screening tool. Assesses for: ● chamber enlargement ● diastolic dysfunction (MV E/A) ● systolic function (LVEF by 3DE) ● subclinical disease (strain) ● contractility via stress echo	Adjunct tool in selected cases. Assesses for: ● chamber enlargement ● systolic function (LVEF) ● epicardial coronary disease (coronary CT)	Adjunct tool in selected cases. Assesses for: ● chamber enlargement ● diastolic dysfunction (LV mass, LV hypertrophy, LA size) ● systolic function (LVEF) ● subclinical disease (strain) ● fibrosis and edema (LGE, T1 mapping, T2 mapping, ECV) ● perfusion via stress cMR	Signs of LVH	Assessment of cardiac output and wedge pressure, which can reveal elevated LV filling pressures in diastolic dysfunction and PVH due to left heart disease
Pericardial Disease	Initial screening tool. Assesses for: ● effusion ● tamponade (diastolic collapse of RV, respirophasic changes across MV and TV, ventricular interdependence) ● constriction (high E velocity, reduced A wave, decreased MV inflow velocity, increased TV velocity)	Pericardial calcifications, density assessment of fluid	Adjunct tool in selected cases. Assesses for: ● effusion ● pericardial thickening ● pericardial edema and inflammation (T2 images, LGE) ● constriction (respiratory flow variation across the mitral valve, septal excursion)	Diffuse ST segment elevations and PR segment depressions, electrical alternans and low voltage in large effusions	End-diastolic equalization of pressures across cardiac chambers in constriction; the stiffened pericardium limits expansion and exerts equal pressure on all chambers (requires concurrent R + LHC)
Arrhythmia	Provides initial assessment of cardiac structure and function in the setting of arrhythmia	N/A	Edema (T2 ratio) and fibrosis (%LGE) may predict ventricular arrhythmias	PVCs, conduction disease, atrial and ventricular arrhythmias	N/A
Strengths	Cheap, widely available. No radiation	Evaluation of underlying etiology of RV dysfunction/PAH (ILD, CTEPH). Not reliant on acoustic windows	Not reliant on acoustic windows. No radiation. Images can be reconstructed in any plane	Cheap, widely available	Gold standard assessment for PH
Limitations	Complex geometry of RV limits evaluation. Operator dependent. Requires adequate acoustic windows. Novel techniques such as STE require additional time, resources, and expertise	Radiation exposure. Limited assessment of hemodynamics. Motion artifact particularly at high heart rates	Limited availability. Expensive. Significant expertise required in acquisition and interpretation. Long examination time. Requires frequent breath holding. Sensitive, but findings often non-specific for SSc and require careful clinical correlation	Not highly sensitive or specific for any disease	Provides no information about morphology. Invasive

*SSc, systemic sclerosis; echo: echocardiography; CT, computed tomography; cMR, cardiovascular magnetic resonance; EKG, electrocardiography; RHC, right heart catheterization; RV, right ventricle; TV, tricuspid valve; E/A; peak velocity in early diastole (E wave) to peak velocity flow in late diastole (atrial contraction, A wave); TAPSE, tricuspid annular plane systolic excursion; FAC, fractional area change; EF, ejection fraction; 3DE, three-dimensional echocardiography; RVSP, right ventricular systolic pressure; LV, left ventricle; ECV, extracellular volume; RVH, right ventricular hypertrophy; PAH, pulmonary arterial hypertension; PASP, pulmonary artery systolic pressure; PA, pulmonary artery; WHO, World Health Organization; MV; mitral valve; LVEF, left ventricular ejection fraction; LGE, late gadolinium enhancement; LVH, left ventricular hypertrophy; PVH, pulmonary venous hypertension; PVC, premature ventricular contractions; ILD, interstitial lung disease; CTEPH, chronic thromboembolic pulmonary hypertension; PH, pulmonary hypertension; STE, speckle-tracking echocardiography.*

Given the abundance of diagnostic options, some nuance is required when approaching a patient with newly diagnosed with SSc. Due to the high prevalence of subclinical cardiac disease in SSc and the low risk and relatively low cost of EKG, BNP or NT-proBNP, and echocardiography, it is reasonable to perform these tests annually. Echocardiography should be performed with Doppler and STE when possible. If further imaging is needed, patients with SSc should be referred to a cardiologist, preferably one with expertise in SSc cardiac disease, who can help guide appropriate and cost-effective management. With that in mind, it is worth noting that that CMR must be interpreted with care. CMR is highly sensitive, but abnormalities found in SSc such as LGE, elevated ECV, and abnormal strain, are not specific for SSc and can be found in a number of diseases (92). Imaging findings must be interpreted while considering the entirety of the clinical context to ensure accurate diagnosis. For this, among other reasons, referral to a cardiologist with SSc experience is ideal.

**FIGURE 4:**
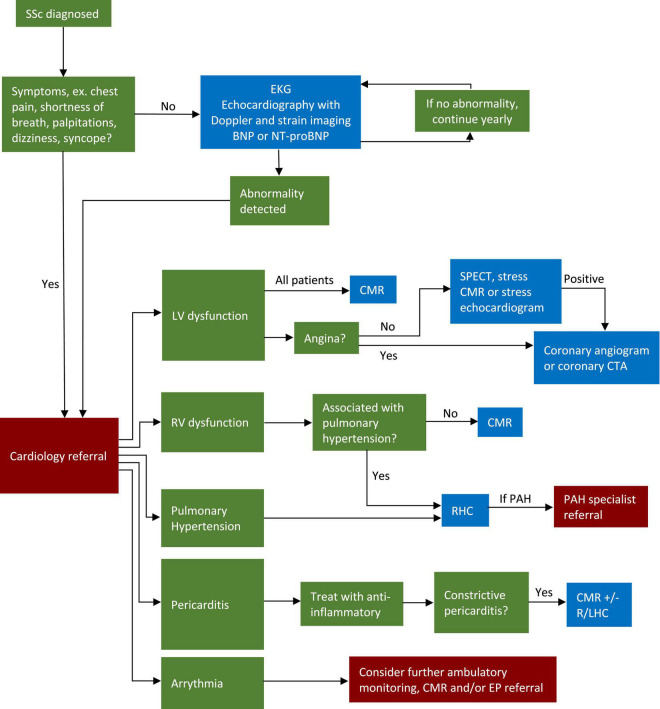
Algorithm for screening and workup of cardiovascular disease in SSc patients. CMR, cardiovascular magnetic resonance; SSc, systemic sclerosis; EKG, electrocardiogram; LV, left ventricle; RV, right ventricle; RHC, right heart catheterization; LHC, left heart catheterization; EP, electrophysiology; CTA, computed tomography angiography; PAH, pulmonary arterial hypertension; SPECT, single photon emission computed tomography.

Of course, the approach will depend on which signs and symptoms are present, as well as the resources and expertise of a given center. With those caveats, we have outlined our approach to cardiac evaluation of the SSc patient in Figure 4. While this approach is based on the current literature, further studies are needed to determine whether this strategy is one that is centered on improvement in patient outcomes.

## Future Directions

There are many novel therapeutics under investigation for use in scleroderma. While none appear specifically targeted toward myocardial involvement, they may impact cardiac disease through anti-inflammatory, anti-fibrotic, vascular/endothelial pathways (93, 94). Cardiovascular imaging has the potential to play an important role in the evaluation of these agents.

Cardiovascular imaging is increasingly being considered as an adjunct to clinical trials. Imaging can help improve patient selection by identifying those with the therapeutic target (i.e., pericardial inflammation or RV systolic dysfunction). It can help determine the baseline distribution of prognostic factors (such as myocardial fibrosis) in treatment and control arms and help shed light on mechanism of disease or response to therapy (95, 96). It can also serve as a measure of efficacy in phase II or phase III trials, helping inform larger randomized controlled trials of hard clinical outcomes. For all these reasons, imaging may play an increasingly important role in the evaluation of therapeutics for SSc.

As an example, a recent meta-analysis identified reduced RV ejection fraction and increased RV volumes as markers of risk for clinical worsening and mortality in patients with PAH; markers that could potentially be used as end points in clinical trials (97). In patients with SSc-PAH, Sato et al. showed that upfront combination therapy with tadalafil and ambrisentan improved CMR-derived LV and RV strain and this correlated with improvement in clinical outcomes, including WHO functional class, 6MWD, NT-proBNP, and invasive hemodynamic markers (38). However, these data were all collected as small case-control, case series, or single-arm studies. Incorporating CMR data into randomized, double-blind, placebo-controlled trials of SSc-PAH therapies with both imaging and clinical outcomes may yield vital information. First, it would help definitively establish the relationship between imaging markers of disease and clinical endpoints. Second, as discussed above, it may help establish meaningful thresholds in CMR markers for clinical monitoring or future trials, as well as mechanistic insights into PAH pathophysiology.

While PAH has served a model for this discussion, one can imagine CMR being incorporated into randomized controlled trials of other SSc-related cardiac pathology. For instance, randomized controlled trials examining treatment effects of novel anti-inflammatories in SSc-related pericarditis may measure recurrence rates and persistence of symptoms as well as CMR markers of pericardial inflammation on follow-up. Trials of novel anti-fibrotics such as pirfenidone in SSc-related cardiac fibrosis may assess arrhythmia burden as well as CMR measures of fibrosis. By including these imaging endpoints, trials can help definitively connect imaging to outcomes, enabling confident use of surrogate endpoints going forward and facilitating clinical monitoring of disease.

Use of artificial intelligence continues to proliferate in imaging generally and it will undoubtedly impact SSc care. Already deep learning techniques are being used to aid in tissue segmentation and identification of fibrosis (98). Given the prominent role fibrosis plays in SSc and particularly arrhythmia risk, automated techniques may allow for earlier identification of at risk individuals. Algorithms are being developed to aid in motion and deformation pattern analysis (98), which may allow for earlier detection of subtle abnormalities, especially in the geometrically complex RV. Finally, learning algorithms combining both clinical and imaging may be helpful in guiding treatment selection and predicting response (98). Given the complexity of the disease and its multiple treatment options, SSc may prove a fertile ground for these types of studies.

## Conclusion

Cardiac complications of SSc are common, varied, and impact heavily on patient outcomes. We have reviewed the evidence supporting the use of cardiac imaging in the evaluation and management of SSc heart disease and have offered our own approach to caring for the SSc patient based on current literature. While cardiac imaging is already foundational in that evaluation, we anticipate it will assume growing importance in the years to come.

## Author Contributions

PG, SH, and TH contributed to the literature review and drafting the manuscript and figures. BF provided editorial revision. All authors contributed to the article and approved the submitted version.

## Conflict of Interest

The authors declare that the research was conducted in the absence of any commercial or financial relationships that could be construed as a potential conflict of interest.

## Publisher’s Note

All claims expressed in this article are solely those of the authors and do not necessarily represent those of their affiliated organizations, or those of the publisher, the editors and the reviewers. Any product that may be evaluated in this article, or claim that may be made by its manufacturer, is not guaranteed or endorsed by the publisher.
